# Supplementary material for: Genetic, natal and spatial drivers of social phenotypes in wild great tits
Source: J Anim Ecol. 2024 Dec 30;94(2):220–32. doi: 10.1111/1365-2656.14234 (PMC11794972; doi:10.1111/1365-2656.14234)
Supplement: Supplementary file 1 — Table S1. Summary table for all model structures detailing the objectives and overall output from the models. Table S2. Variance components from 5 animal models (with various combinations of random effects used) detailing the estimates (standard error). Table S3. Repeatability and narrow‐sense heritability estimates for social network traits within‐years (2011, 2012 and 2013) and between year using two models; V p = V ID + V r shown as V ID, and V p = VID + V A + V r shown as V ID + V A. Table S4. Variance components from 9 animal models (with various combinations of random effects used) detailing the estimates. [file JANE-94-220-s001.docx]

**Supplementary Information for ‘Genetic, natal, and spatial drivers of social phenotypes in wild great tits’**

**Table 1.** Summary table for all model structures detailing the objectives and overall output from the models.

| **Response variable** Number of individuals (observations) | ***Model*** (Random effects incorporated) | **Model Structure** | **Objective of Model** | **Overall output** |
| --- | --- | --- | --- | --- |
| **Group Size**  2011 - 1,085 (343,458 observations)  2012 - 720 (244,064 observations)  2013 - 789 (204,296 observations)  All 3 winters - 1,823 individuals (791,818 observations) – *Models have additional fixed effect (winter year)* | ***Model 1***  (individual identity) | *V_P_ = V_ID_ + V_R_* | Calculate individual repeatability using R_ID_ = *V_ID_/ V_R_* | Proportion of group size variation attributable to individual identity |
|  | ***Model 2***  (individual identity, relatedness matrix) | *V_P_ = V_ID_ + V_A_ + V_R_* | Calculate R_ID_ = (*V_ID_ + V_A_)/ V_R_*  and heritability as h^2^ = V_A_/V_P_ | Heritable component of group size variation |
|  | ***Model 3***  (logger identity) | *V_P_ = V_LOG_ + V_R_* | Calculate logger repeatability, R_LOG_ = *V_LOG_ / V_P_* | Consistency of group size at various spatial locations (loggers) |
|  | ***Model 4***  (individual identity, logger identity) | *V_P_ = V_ID_ + V_LOG_ + V_R_* | Calculate R_LOG_ = *V_LOG_ / V_P_* and R_ID_ = *V_ID_/ V_R_* | Proportion of group size variation attributable to individual identity or spatial location |
|  | ***Model 5***  (individual identity, logger identity, relatedness matrix) | *V_P_ = V_ID_ + V_LOG_ + V_A_ + V_R_* | Calculate R_ID_ = (*V_ID_ + V_A_)/ V_R_*  , h^2^ = V_A_/V_P_ and R_LOG_ = *V_LOG_ / V_P_* | Proportion of group size variation attributable to individual identity, spatial location, and genetic factors |
| Social Network-derived phenotypes (**mean group size, degree, weighted degree, centrality, weighted centrality, betweenness**)  2011 - 1,085 (9855 observations)  2012 - 720 (6538 observations)  2013 - 789 (7348 observations)  All 3 winters - 1,823 individuals (23741 observations) – *Models have additional fixed effect (winter year)* | ***Model 1***  (individual identity) | *V_P_ = V_ID_ + V_R_* | Calculate individual repeatability using R_ID_ = *V_ID_/ V_R_* | Proportion of trait variation attributable to individual identity |
|  | ***Model 2***  (individual identity, relatedness matrix) | *V_P_ = V_ID_ + V_A_ + V_R_* | Calculate R_ID_ = (*V_ID_ + V_A_)/ V_R_*  and heritability as h^2^ = V_A_/V_P_ | Heritable component of trait variation |
| **NATAL EFFECTS**  **Group size**  All 3 winters - 938 (401,915 observations)  **Degree**  All 3 winters - 938 (12052 observations)  *All models have winter year as fixed effect* | ***Model a***  (individual identity) | *V_P_ = V_ID_ + V_R_* | Calculate individual repeatability using R_ID_ = *V_ID_/ V_R_* | Proportion of trait variation attributable to individual identity |
|  | ***Model b***  (individual identity, relatedness matrix) | *V_P_ = V_ID_ + V_A_ + V_R_* | Calculate R_ID_ = (*V_ID_ + V_A_)/ V_R_*  and heritability as h^2^ = V_A_/V_P_ | Heritable component of trait variation |
|  | ***Model c***  (individual identity, natal section) | *V_P_ = V_ID_ +* ***V_SECT_*** *+ V_R_* | Calculate *V_SECT_ / V_P_* | Proportion of trait variation attributable to natal section |
|  | ***Model d***  (individual identity, brood identity) | *V_P_ = V_ID_ +* ***V_BI_*** *+ V_R_* | Calculate *V_BI_ / V_P_* | Proportion of trait variation attributable to brood identity |
|  | ***Model e***  (individual identity, brood identity, natal section) | *V_P_ = V_ID_ + V_SECT_ + V_BI_ + V_R_* | Calculate *V_SECT_ / V_P_ and V_BI_ / V_P_* | Proportion of trait variation attributable to both natal effects (brood identity and natal section) |
|  | ***Model f***  (individual identity, relatedness matrix, brood identity, natal section, **logger identity**) | *V_P_ = V_ID_ + V_A_ + V_SECT_ + V_BI_ + V_LOG_ + V_R_*  ** V_LOG_ removed for degree models* | Calculate *V_SECT_ / V_P_ and V_BI_ / V_P_*  Along with h^2^ = V_A_/V_P_  and R_LOG_ = *V_LOG_ / V_P_* | Proportion of trait variation attributable to both natal effects (brood identity and natal section) + heritable component of trait and effect of spatial location |
|  | ***Model g***  (individual identity, relatedness matrix, natal spatial matrix) | *V_P_ = V_ID_ + V_A_ + V_SPATIAL_ + V_R_* | Calculate *V_SPATIAL_ / V_P_*  and h^2^ = V_A_/V_P_ | Proportion of trait variation attributable to natal spatial proximity |
|  | ***Model h***  (individual identity, relatedness matrix, natal environmental similarity matrix) | *V_P_ = V_ID_ + V_A_ + V_ENVSIM_ + V_R_* | Calculate *V_ENVSIM_ / V_P_*  and h^2^ = V_A_/V_P_ | Proportion of trait variation attributable to natal environmental similarity |
|  | ***Model i***  individual identity, relatedness matrix, natal spatial matrix, natal environmental similarity matrix) | *V_P_ = V_ID_ + V_A_ + V_SPATIAL_+ V_ENVSIM_ + V_R_* | Calculate *V_SPATIAL_ / V_P_, V_ENVSIM_ / V_P_*  and h^2^ = V_A_/V_P_ | Proportion of trait variation attributable to natal environmental similarity, natal spatial proximity and the heritable component of trait |

**Table 2.** Variance components from 5 animal models (with various combinations of random effects used) detailing the estimates (standard error). V_ID_ = focal individual permanent environment effect, V_A_ = additive genetic effect, V_LOG_ = spatial effect of logger/feeder, V_R_ = residual variance, V_P_ = total phenotypic variance (sum of all variance components), R_ID_ = Repeatability of group size for focal individuals given as proportion of V_ID_ or V_ID_ +V_A_ to V_P_, R_LOG_= Repeatability of group size at loggers given as proportion of V_LOG_ to V_P_, h^2^ = narrow-sense heritability as proportion of V_A_ to V_P_, χ2 term to test the significance of the random effect with p-value.

| **Model** | **Year** | **2011** | **2012** | **2013** | **All 3 (FE=year)** |
| --- | --- | --- | --- | --- | --- |
| Random effect = ID  (*Model 1)* | V_ID_ | 4.548 (0.206) | 5.999 (0.328) | 2.891 (0.157) | 4.205 (0.147) |
|  | V_R_ | 19.028 (0.045) | 13.739 (0.039) | 11.153 (0.034) | 15.99 (0.025) |
|  | V_P_ | 23.576 | 19.738 | 14.044 | 20.195 |
|  | R_ID_ | **0.192** (0.007) | **0.303** (0.011) | **0.205** (0.008) | **0.209** (0.005) |
| Random effect = ID + pedigree  (*Model 2*) | V_ID_ | 4.548 (0.206) | 5.673 (0.498) | 2.589 (0.221) | 4.159 (0.187) |
|  | V_A_ | 5.948 x 10^-7^ | 0.392 (0.489) | 0.366 (0.225) | 0.06 (0.153) |
|  | V_R_ | 19.028 (0.045) | 13.739 (0.039) | 11.153 (0.034) | 15.99 (0.025) |
|  | V_P_ | 23.576 | 19.804 | 14.108 | 20.209 |
|  | R_ID_ | 0.192 (0.007) | 0.306 (0.012) | 0.209 (0.009) | 0.208 (0.005) |
|  | h^2^ | 2.523 x 10^-8^ (2.26 x 10^-10^) | 0.019 (0.024) | 0.026 (0.015) | 0.003 (0.007) |
| Random effect = logger  (*Model 3*) | V_LOG_ | 7.230 (1.280) | 4.920 (0.851) | 3.842 (0.682) | 4.899 (0.866) |
|  | V_R_ | 17.523 (0.042) | 11.718 (0.033) | 9.901 (0.03) | 15.094 (0.023) |
|  | V_P_ | 24.753 | 16.638 | 13.743 | 19.993 |
|  | R_LOG_ | 0.292 (0.036) | 0.296 (0.036) | 0.279 (0.035) | 0.245 (0.032) |
| Random effect = ID + logger  (*Model 4*) | V_ID_ | 1.300 (0.064) | 0.813 (0.049) | 0.428 (0.027) | 1.767 (0.065) |
|  | V_LOG_ | 8.442 (1.498) | 5.996 (1.06) | 4.191 (0.746) | 5.624 (0.995) |
|  | V_R_ | 16.804 (0.040) | 11.235 (0.032) | 9.604 (0.03) | 14.13 (0.022) |
|  | V_P_ | 26.546 | 18.044 | 14.223 | 21.521 |
|  | R_ID_ | 0.048 (0.003) | 0.045 (0.003) | 0.030 (0.002) | 0.082 (0.004) |
|  | R_LOG_ | 0.318 (0.038) | 0.332 (0.039) | 0.294 (0.037) | 0.261 (0.034) |
|  | χ2 (logger term) | 43401.13 (p < 0.001) | 49904.64 (p < 0.001) | 31392.41 (p < 0.001) | 98701.52 (p < 0.001) |
| Random effect = ID + logger + pedigree  (*Model 5*) | V_ID_ | 1.209 (0.086) | 0.813 (0.049) | 0.428 (0.027) | 1.735 (0.083) |
|  | V_LOG_ | 8.445 (1.498) | 5.996 (1.064) | 4.191 (0.746) | 5.624 (0.995) |
|  | V_A_ | 0.123 (0.086) | 2.2 x 10^-7^ | 1.5 x 10^-7^ | 0.041 (0.070) |
|  | V_R_ | 16.804 (0.040) | 11.233 (0.032) | 9.604 (0.03) | 14.130 (0.022) |
|  | V_P_ | 26.581 | 18.042 | 14.223 | 21.53 |
|  | R_ID_ | 0.050 (0.003) | 0.045 (0.003) | 0.030 (0.002) | 0.082 (0.004) |
|  | R_LOG_ | 0.317 (0.038) | 0.332 (0.038) | 0.294 (0.037) | 0.261 (0.034) |
|  | h^2^ | 0.004 (0.003) | 1.22 x 10^-8^ (6.9 x 10^-10^) | 1.06 x 10^-8^ (5.5 x 10^-10^) | 0.001 (0.003) |
|  | χ2 (logger term) | 43403.73 (p < 0.001) | 49903.82 (p < 0.001) | 31389.02 (p < 0.001) | 98701.74 (p < 0.001) |

**Table 3.** Repeatability & narrow-sense heritability estimates for social network traits within-years (2011, 2012, and 2013) and between-year using two models; V_p_ = V_ID_ + V_r_ shown as V_ID_, and V_p_ = V_ID_ + V_A_ + V_r_ shown as V_ID_ + V_A_. V_ID_ = focal individual permanent environment effect, V_A_ = additive genetic effect, V_R_ = residual variance, V_P_ = total phenotypic variance, R_ID_ = Repeatability of group size for focal individuals given as proportion of V_ID_ or V_ID_ +V_A_ to V_P_, h^2^ = narrow-sense heritability as proportion of V_A_ to V_P_.

| **Trait** | **Model** | **Estimate** | **2011** | **2012** | **2013** | **Between-year** |
| --- | --- | --- | --- | --- | --- | --- |
| Mean group size | V_ID_ | R | 0.379 (0.013) | 0.586 (0.015) | 0.545 (0.015) | 0.423 (0.01) |
|  | V_ID_ + V_A_ | R | 0.379 (0.013) | 0.591 (0.015) | 0.549 (0.015) | 0.425 (0.01) |
|  |  | h^2^ | 4.42 x 10^-8^ (9.65 x 10^-10^) | 0.062 (0.051) | 0.063 (0.041) | 0.0109 (0.017) |
| Degree | V_ID_ | R | 0.379 (0.013) | 0.574 (0.015) | 0.493 (0.015) | 0.413 (0.01) |
|  | V_ID_ + V_A_ | R | 0.379 (0.013) | 0.577 (0.016) | 0.496 (0.016) | 0.413 (0.01) |
|  |  | h^2^ | 5.3 x 10^-8^ (1.16 x 10^-9^) | 0.049 (0.049) | 0.034 (0.036) | 5.89 x 10^-8^ (10^-9^) |
| Weighted Degree | V_ID_ | R | 0.375 (0.013) | 0.554 (0.0157) | 0.534 (0.0151) | 0.416 (0.01) |
|  | V_ID_ + V_A_ | R | 0.379 (0.013) | 0.557 (0.0136) | 0.542 (0.016) | 0.417 (0.010) |
|  |  | h^2^ | 5.1 x 10^-8^ (1.11 x 10^-9^) | 0.043 (0.049) | 0.106 (0.042) | 0.0108 (0.016) |
| Weighted Centrality | V_ID_ | R | 0.086 (0.007) | 0.259 (0.014) | 0.046 (0.007) | 0.097 (0.005) |
|  | V_ID_ + V_A_ | R | 0.09  (0.008) | 0.265 (0.015) | 0.054 (0.008) | 0.099 (0.005) |
|  |  | h^2^ | 0.02  (0.01) | 0.048 (0.029) | 0.043 (0.012) | 0.010 (0.006) |
| Centrality | V_ID_ | R | 0.126 (0.009) | 0.365 (0.016) | 0.136 (0.01) | 0.169 (0.007) |
|  | V_ID_ + V_A_ | R | 0.131 (0.009) | 0.374 (0.017) | 0.141 (0.011) | 0.172 (0.007) |
|  |  | h^2^ | 0.028 (0.012) | 0.103 (0.035) | 0.036 (0.018) | 0.022 (0.009) |
| Between-ness | V_ID_ | R | 0.0551 (0.006) | 0.0423 (0.008) | 0.076 (0.008) | 0.049 (0.004) |
|  | V_ID_ + V_A_ | R | 0.057 (0.007) | 0.042 (0.008) | 0.077 (0.009) | 0.050 (0.004) |
|  |  | h^2^ | 0.0123 (0.009) | 1.6 x 10^-8^ (2.97 x 10^-10^) | 0.007 (0.012) | 0.008 (0.004) |

**Table 4.** Variance components from 9 animal models (with various combinations of random effects used) detailing the estimates. V_ID_ = focal individual permanent environment effect, V_A_ = additive genetic effect, V_SECT_ = natal section, V_BI_ = brood identity, V_LOG_ = spatial effect of logger/feeder, V_SPAT_ = spatial proximity matrix, V_ENV_ = natal environmental similarity matrix, V_R_ = residual variance, χ2 term to test the significance of the random effect with p-value.

| **Response Variable** | | **Group size** | | **Degree** | |
| --- | --- | --- | --- | --- | --- |
| **Model** | Variance Component | Estimate | Proportion (Relative to V_P_) | Estimate | Proportion (Relative to V_P_) |
| **Model a** | V_ID_ | 4.024 | 0.206 | 85.486 | 0.416 |
|  | V_R_ | 15.479 | 0.793 | 120.059 | 0.584 |
| **Model b** | V_ID_ | 3.430 | 0.175 | 77.121 | 0.375 |
|  | V_A_ | 0.615 | 0.031 | 8.510 | 0.041 |
|  | V_R_ | 15.479 | 0.792 | 120.076 | 0.584 |
| **Model c** | V_ID_ | 3.963 | 0.200 | 79.889 | 0.388 |
|  | V_SECT_ | 0.166 | 0.008 | 4.322 | 0.021 |
|  | V_R_ | 15.590 | 0.790 | 121.443 | 0.591 |
|  | χ2 (p-value) | 54974.63 (p < 0.001) | | 3620.161 (p < 0.001) | |
| **Model d** | V_ID_ | 3.621 | 0.185 | 76.602 | 0.372 |
|  | V_BI_ | 0.431 | 0.022 | 8.933 | 0.043 |
|  | V_R_ | 15.497 | 0.792 | 120.219 | 0.584 |
|  | χ2 (p-value) | 17366.51 (p < 0.001) | | 1037.847 (p < 0.001) | |
| **Model e** | V_ID_ | 3.668 | 0.186 | 75.562 | 0.367 |
|  | V_SECT_ | 0.162 | 0.008 | 4.229 | 0.021 |
|  | V_BI_ | 0.301 | 0.015 | 4.426 | 0.022 |
|  | V_R_ | 15.590 | 0.790 | 121.441 | 0.590 |
|  | χ2 (p-value) | 54976.72 (p < 0.001) | | 3621.109 (p < 0.001) | |
| **Model f** | V_ID_ | 1.586 | 0.075 | 75.562 | 0.367 |
|  | Va | 0.012 | 0.0006 | 6.67E-06 | 3.24E-08 |
|  | V_SECT_ | 0.003 | 0.0001 | 4.229 | 0.021 |
|  | V_BI_ | 0.132 | 0.006 | 4.426 | 0.022 |
|  | V_LOG_ | 5.432 | 0.260 | 0.000 | 0.000 |
|  | V_R_ | 13.710 | 0.656 | 121.441 | 0.590 |
|  | χ2 (p-value) | 49825.15 (p < 0.001) – logger term | | NA | |
| **Model g** | V_ID_ | 3.790 | 0.191 | 78.450 | 0.366 |
|  | V_A_ | 0.008 | 0.0004 | 1.13E-05 | 5.3E-08 |
|  | V_SPAT_ | 0.538 | 0.027 | 16.051 | 0.075 |
|  | V_R_ | 15.479 | 0.781 | 120.098 | 0.560 |
|  | χ2 (p-value) | 21.19449 (p < 0.001) | | 37.65627 (p < 0.001) | |
| **Model h** | V_ID_ | 3.602 | 0.182 | 79.13 | 0.371 |
|  | V_A_ | 0.270 | 0.013 | 2.94E-05 | 1.38E-07 |
|  | V_ENV_ | 0.380 | 0.0192 | 13.731 | 0.064 |
|  | V_R_ | 15.479 | 0.784 | 120.07 | 0.563 |
|  | χ2 (p-value) | 9.851628 (p < 0.001) | | 27.69689 (p < 0.001) | |
| **Model i** | V_ID_ | 3.790 | 0.191 | 78.450 | 0.365 |
|  | Va | 0.008 | 0.0004 | 1.128E-05 | 5.258E-08 |
|  | V_SPAT_ | 0.538 | 0.0271 | 16.051 | 0.0747 |
|  | V_ENV_ | 1.865E-07 | 9.41E-09 | 8.08E-06 | 3.76E-08 |
|  | V_R_ | 15.479 | 0.781 | 120.097 | 0.559 |
|  | χ2 (p-value) | 21.19449 (p < 0.001) | | 37.65627 (p < 0.001) | |
